# Communication Strategies Used in Primary Progressive Aphasia: A Scoping Review

**DOI:** 10.1177/14713012251356588

**Published:** 2025-07-09

**Authors:** Inês Cadório, Daniela Vieira

**Affiliations:** 1 RISE-Health, Escola Superior de Saúde Fernando Pessoa, Fundação Ensino e Cultura Fernando Pessoa, Porto, Portugal; 2 Instituto de Investigação, Inovação e Desenvolvimento (FP-I3ID), Unidade de Investigação de Ciências Biomédicas e da Saúde Biomedical (FP-BHS), Universidade Fernando Pessoa, Porto, Portugal; 3 Escola Superior de Saúde Fernando Pessoa (ESS-FP), Porto, Portugal

**Keywords:** primary progressive aphasia, communication strategies, compensatory strategies, augmentative and alternative communication, communication partners

## Abstract

Primary progressive aphasia describes a group of language-led dementias that affects an individual’s ability to speak, comprehend, read and write. While post-stroke aphasia has been the subject of extensive research, particularly in the domain of communication strategies, the same level of research interest has not been observed in PPA. This is indicative of a significant knowledge gap regarding effective communication strategies in PPA. The present review aims to outline the current research evidence related to the range of communication strategies employed within PPA individuals and their effectiveness. A series of studies were considered, encompassing patients diagnosed with PPA, their spouses and/or other communication partners, provided that data on the utilization and training of communication strategies was addressed. A total of 26 non-experimental and experimental studies met the inclusion criteria. Communication strategies retrieved varied from verbal, to non-verbal, environmental modification strategies and partner adaptation strategies. The findings demonstrate that assistive augmented communication strategies have a positive impact on communication interactions. Furthermore, the multiple use of different modalities ensures the optimal conveyance of messages. Consequently, the degree of independence exhibited by the subjects increases, as does their overall quality of life.

## Introduction

In recent decades, there has been a growing interest in neurodegenerative conditions due to the irreversibility of symptoms, the lack of effective treatments, and the associated social and economic burdens (Hung et al., 2010). Individuals with neurodegenerative conditions require substantial assistance with activities of daily living (ADLs), including eating, drinking, dressing, bathing, personal hygiene, use of the toilet, and transportation (Maresova et al., 2020).

Frontotemporal lobar degeneration (FTLD) and Alzheimer’s disease represent examples of early-onset dementias (Jarmolowicz et al., 2014). Both FTLD and Alzheimer’s disease frequently present with primary progressive aphasia (PPA) (Bergeron et al., 2018). Clinicopathological correlations in PPA, thus, reflect the contributory role of Pick’s disease and other tauopathies, such as corticobasal degeneration, progressive supranuclear palsy, corticobasal degeneration, motor features of amyotrophic lateral sclerosis that are associated with TDP-43, dementia with Lewy bodies or proteinopathies (Grossman, 2010).

PPA is diagnosed when there are prominent deficits in language functions that have a detrimental impact on ADLs. In the initial two-year period, aphasia is typically accompanied by relatively preserved cognitive abilities (Gorno-Tempini et al., 2011). PPA can be subdivided into three main variants, which are distinguished by their distinctive speech and language profiles, patterns of brain atrophy, and underlying pathologies (Butts et al., 2015). This means that each PPA clinical variant is associated with a typical and most frequent cognitive, neuroimaging, and neuropathological profile (Spinelli et al., 2017).

Semantic variant of PPA (svPPA) is characterized by impaired confrontation naming, impaired single-word comprehension, impaired object knowledge and surface dyslexia or dysgraphia. In svPPA, a predominant anterior temporal lobe atrophy and/or anterior temporal hypoperfusion or hypometabolism is observed on single-photon emission computed tomography (SPECT) or positron emission tomography (PET) (Gorno-Tempini et al., 2011). Research conducted in the clinical and pathological domain has demonstrated a link between the presence of svPPA and the manifestation of TAR DNA-binding protein 43 (TDP-43) pathologies (Bergeron et al., 2018). In non-fluent or agrammatic variant (nfvPPA), agrammatism in language production is evident, comprehension of syntactically complex sentences is impaired, and apraxia of speech may manifest. Neuroimaging reveals a predominant left posterior fronto-insular atrophy on magnetic resonance imaging (MRI) or left posterior fronto-insular hypoperfusion or hypometabolism on SPECT or PET scans (Gorno-Tempini et al., 2011). Corticobasal degeneration and Pick’s disease have been demonstrated to be significantly associated with nfvPPA (Bergeron et al., 2018). In logopenic variant PPA (lvPPA), individuals experience difficulties in retrieving words, as well as repeating sentences and phrases. Additionally, speech errors are observed in spontaneous speech and naming tasks. MRI of individuals with lvPPA reveals predominant atrophy in the left posterior perisylvian or parietal regions, as well as reduced perfusion or metabolic activity in these regions, as indicated by SPECT or PET scans (Gorno-Tempini et al., 2011). Amyloid pathology has been identified as a frequently underlying pathology in cases of lvPPA (Bergeron et al., 2018). As previously stated, communication is the most significantly affected ability due to the gradual deterioration of language skills, particularly in word-finding and spelling (Mesulam et al., 2012). To circumvent communication breakdowns due to the damaged system, intact systems or skills are recruited for the purpose of training compensatory strategies (Hopper, 2007). A further objective of behavioural interventions may be to assist communication partners in providing more effective support for communication interactions (O’Rourke et al., 2018).

Volkmer and collaborators (Volkmer et al., 2020) emphasised the pivotal role of individuals’ existing communication strategies and the practice of these strategies with caregivers in the context of functional communication interventions. One might posit that communication strategies represent the way individuals with aphasia and their respective conversation partners successfully overcome the communication barriers imposed by aphasia (Beckley et al., 2017). Verbal and non-verbal communication strategies may include the use of circumlocution, keywords, signing, gesturing, pantomime, drawing, writing, conversation continuers (e.g., ‘mm hum’), communication charts, and high- or low-tech communication aids (Beeke et al., 2015; Hux et al., 2008; Sandt-Koenderman, 2004; Wilkinson et al., 2011). Prior to offering a concise characterisation of high- and low-tech communication aids, it is imperative to comprehend the concept of Augmentative and Alternative Communication (AAC). This term encompasses a diverse array of tools, techniques, strategies, environmental and partner adaptations, and behavioural modifications. The purpose of AAC is to compensate for the loss of speech and language function (Fried-Oken et al., 2010). The range of communication aids extends from low-tech to high-tech AAC. Low-tech AAC refers to communication techniques that do not rely on electronic or computerized devices, such as using pen and paper, alphabet boards, communication books, alerting systems, writing messages, drawing pictures, pointing words or basic signaling tools to help individuals express themselves (Fried-Oken et al., 2015). In contrast, high-tech AAC refers to technologically advanced communication tools and systems (Russo et al., 2017), such as speech-generating devices (Fried-Oken et al., 2015).

Regarding PPA, there is a paucity of knowledge regarding the type of communication strategies employed in conversations and/or trained during the intervention period. Furthermore, the extent to which these strategies are effective is also unclear. Accordingly, the objective of this exploratory study is to systematically map the breadth of literature on communication strategies used and/or trained in the PPA population, identify key concepts, theories, sources of evidence and knowledge gaps.

## Method

To ascertain whether any existing reviews addressed communication strategies and/or training in PPA, a brief search of PROSPERO, the Cochrane Database of Systematic Reviews and JBI Evidence Synthesis was conducted. No reviews were identified on this topic. Prior to the commencement of the study, a protocol was devised which set out the objective, search strategy, eligibility criteria, procedures for evidence collection and data extraction. The protocol was duly registered with Figshare (reference: 10.6084/m9.figshare.25465222). The process was informed by the Preferred Reporting Items for Systematic Review and Meta-Analysis Extension of Scoping Reviews Checklist (PRISMA-ScR) throughout, with decisions made in accordance with its tenets (Tricco et al., 2018) (Annex I). Furthermore, the Joanna Briggs Institute recommendations were adhered to enhance the methodological quality of the study (Pollock et al., 2023). Two researchers were involved in the review. The following section outlines the methodology employed in the review process, including the search strategy, eligibility criteria, study selection, data extraction, reviewer reliability, data analysis and presentation.

### Eligibility Criteria (Participants, Concept, Context, Types of Sources)

In accordance with the recommendations, the authors of the scoping review employed the Population, Concept and Context (PCC) framework as a guide to determine the eligibility criteria (Peters et al., 2020).

#### Participants

This review considered studies that included persons with PPA (pwPPA) or any subtype of PPA at any time post-onset, irrespective of whether they were receiving treatment. Furthermore, studies that encompassed patients’ spouses or other relevant communication partners (e.g., health professionals) were also included in the review. Conversely, studies in which participants exhibited post-stroke aphasia or aphasia subsequent to brain injury were excluded, as were studies in which the participants’ type of aphasia was not specified.

#### Concept

This review encompasses studies that investigate the use and/or training of communication strategies amongst pwPPA and their significant communication partners. The authors employed the definition proposed by Simmons-Mackie and Damico (Simmons-Mackie & Damico, 1997), which defines a communication strategy as a novel or expanded communicative behaviour, whether spontaneously acquired or repeatedly employed, that is used to overcome a communication barrier, in order to meet transactional and interactional communicative needs.

The studies of interest comprised an array of communication strategies, encompassing both verbal and non-verbal modalities. The objective of the reviewers was to identify a comprehensive range of communication strategies that are part of the everyday lives of PPA individuals and those which are employed by conversation partners in dyadic or group conversations.

#### Context

The scoping review included studies from a range of countries, without imposing any geographical limitations for exclusion purposes. Furthermore, international studies conducted in a diverse array of settings were included for review, including healthcare facilities (such as primary care, hospital-based, and private practice settings), academic institutions (such as university departments), and community-based organizations. The studies included in the review were permitted to report data collected in person or in other ways, including through video conferencing.

#### Types of Sources

To ensure the quality and rigour of the sources included in this scoping review, only articles in their full version, published in peer-reviewed and indexed journals, were accepted. In addition, books, book chapters and theses were included in the review, while conference proceedings, opinion pieces and commentaries were excluded. Although studies conducted in disparate countries were accepted for consideration, only those written in English were accepted.

Only studies published from 2001 onwards were considered, as the evidence-based diagnostic criteria for PPA were established by Mesulam (Mesulam, 2001), thereby facilitating a common terminology within the scientific community.

Selected studies could employ a variety of study designs, including quantitative, qualitative, or combined designs. In addition to experimental study designs, quasi-experimental study designs were also considered in this scoping review. These included randomized controlled trials, non-randomized controlled trials, before-after studies, case series and single-case studies. Moreover, observational studies, including prospective and retrospective cohort studies, case-control studies, cross-sectional studies, and descriptive studies, were also included. Secondary literature, including reviews of any kind (e.g., systematic reviews, scoping reviews, narrative reviews), was also considered.

### Search Strategy

An initial limited search was conducted in two different databases: Medline and Web of Science. Grounded in documented authors expertise in writing about PPA and their prior experience conducting narrative and systematic reviews (Cadório et al., 2017, 2019), along with the results of the initial pilot search, keywords were established to perform a new search on the selected databases. A systematic search of the aphasia literature included the following databases: PubMed, Scopus, Medline, LILACS, Web of Science, PsychINFO, Educational Resources Information Center (ERIC) and Portuguese National Open Access Scientific Repository (RCAAP). Accordingly, the advanced search was conducted in accordance with the specific conventions of the respective database, including the mapping and utilization of multiple search fields and filters. The search was constructed using Medical Subject Headings (MeSH), which were selected with great care to serve the specific purposes of this study. A comprehensive controlled vocabulary enabled the retrieval of relevant literature, including journal articles, theses, books and/or book chapters in the life sciences, particularly in the field of communication disorders resulting from neurodegenerative conditions. The search strategy entailed the utilization of keywords and index terms embedded within the titles and abstracts of pertinent articles. The following query was implemented: [(‘primary progressive aphasia’ OR ‘semantic dementia’ OR ‘non-fluent progressive aphasia’ OR ‘logopenic aphasia’ OR ‘progressive language’ OR ’language variant FTD’)] AND [(‘communication strategies’ OR ‘communication strategy’ OR ‘communication strategies’ OR ‘communication strategy’ OR ‘compensatory strategies’)].

Following the completion of the advanced search, the reference lists of the articles included in the review were examined to inform the subsequent search strategy.

### Selection of Studies

All records retrieved from the selected databases were imported into Mendeley Reference Manager V1.19.8, and any duplicates were removed. Subsequently, the title and abstracts were evaluated independently by the authors in accordance with the established eligibility criteria. At this juncture, studies were excluded if the year of publication was prior to 2001, if the study only reported a research protocol, or if communication strategies used in PPA were not described in detail. Studies were also excluded, if it was not clear what type of aphasia participants had, if the participants exhibited post-stroke aphasia or aphasia following acquired brain injury. Then, the full text studies were subjected to independent analysis in accordance with the pre-established eligibility criteria, and the reference lists were consulted for additional information. Studies were excluded if they did not comply with the established PCC criteria. The reasons for exclusion are detailed in Appendix 1. To evaluate the reliability of the selected studies, inter-rater agreement was calculated, resulting in a value of 92%. The studies that were the subject of disagreement were discussed by the reviewers and a resolution was reached. A consensus was reached regarding the studies that were ultimately included in the review.

### Data Extraction and Synthesis

The first and second authors developed a data extraction tool through which information deemed pertinent to the review was documented. The data entries encompassed the following information: (1) information about the study - (i) authors and year of publication, (ii) study location, (iii) type of study, (iv) sample size; (v) aim of the study; (2) information about participants - (vi) individuals’ diagnosis; and (3) information about the results - (vii) communication strategies used and/or trained, (viii) study outcomes. The authors created the data extraction tool in alignment with the JBI recommendations for scoping reviews (Peters et al., 2024). In instances where data was lacking or supplementary information was required, the authors of the studies were contacted to request the necessary data.

### Data Analysis and Presentation

Two separate reviewers undertook independent analysis of the selected studies. The results of the data extraction were summarized and presented in tables and figures, thus facilitating reading. Furthermore, a narrative summary of the results was provided to respond to the research question: what communication strategies are employed and/or taught in the context of PPA?

## Results

### Search Results

**Figure 1.**
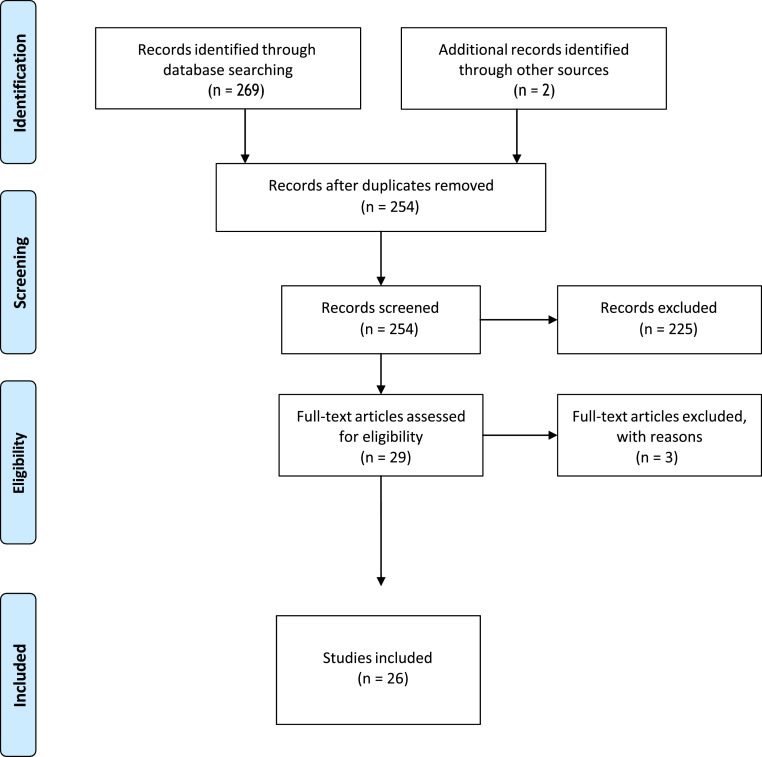
Prisma Flow Diagram of the Scoping Review Process

A search of the databases was conducted between February and May of 2024, yielding a total of 269 citations. Of these, 9 were found in PubMed, 221 in Scopus, 1 in Web of Science, 3 in Medline, 1 in LILACS, 0 in PsychINFO, 24 in ERIC, and 10 in RCAAP. A further two records were identified through searches of grey literature. Following the removal of duplicates, 254 records remained. During the screening phase, a further 225 studies were excluded. Following a full-text eligibility check of the remaining 29 articles, three further studies were excluded. Thus, 26 studies qualified for inclusion in this review. The results of the search conducted for this review are presented in Figure 1, in the form of a PRISMA diagram (Tricco et al., 2018).

Of the 26 studies deemed eligible for inclusion, 10 were non-experimental, while the remaining 16 were experimental. The non-experimental studies were comprised of eight reviews and two overviews. In contrast, the experimental studies comprised seven case reports, three case series, four comparison-group studies and two randomized controlled trials. Regarding the experimental studies, one was conducted in Brazil, one in Portugal, ten in the United States of America (USA), one in Canada, four in the United Kingdom (UK), and two in Australia.

### Participants Characteristics

The participants included in the experimental studies were diagnosed with PPA (*N* = 138), svPPA (*N* = 12), nfvPPA (*N* = 18), lvPAA (*N* = 16), mixed PPA (*N* = 4), Alzheimer’s disease (*N* = 30), mild cognitive impairment (*N* = 1), Communication Partners (CPs) (*N* = 147), and healthy controls (*N* = 10). Most participants were diagnosed with PPA, without a specified subtype. The mean sample size across experimental studies was 20 participants per study. The experimental studies included a total of 386 participants, comprising 67 females and 49 males, with a mean age of 67.7 years. Four studies did not provide information regarding the sex of the individuals, and three studies failed to include data on the age of the participants. The non-experimental studies encompassed pwPPA, classical Alzheimer’s disease, neurocognitive disorders, and frontotemporal dementia.

### Communication Strategies

The review identified a wide range of strategies that can be employed to facilitate effective communication between pwPPA and CPs. The authors have categorized the strategies into four distinct groups: verbal communication strategies, non-verbal communication strategies (Beeke et al., 2015; Hux et al., 2008; Sandt-Koenderman, 2004; Wilkinson et al., 2011), environmental modification strategies (Hinshelwood et al., 2016) and partner adaptation strategies (Fried-Oken et al., 2015).

Verbal communication strategies may be classified in a hierarchical manner, with the least assistive strategies positioned at one end of the spectrum, serving individuals with less severe impairments, and the most assistive strategies at the other end, serving individuals with more severe impairments. Examples of verbal communication strategies used in mild PPA are e-mail (Davies & Howe, 2019; Mooney et al., 2022), using an alternative word when the target word is not available, giving additional information around a person’s identity when unable to generate the name (Volkmer, Walton, et al., 2023), using self-cueing strategies to improve word access (semantic, orthographic or phonological) (Grasso et al., 2019), using voice recognition technology to write (Rogalski & Khayum, 2018), requesting assistance from the partner for specific information or lexical retrieval (Taylor et al., 2014). Conversely, the following represent illustrative examples of the verbal strategies deployed in cases of severe PPA: the use of the term “thing” when the target word is not available (Douglas, 2021), the use of a communication wallet (Mooney, Beale, et al., 2018; Rogalski & Khayum, 2018), the use of yes/no eye blinks (Fried-Oken et al., 2015), the use of remnants (e.g., theatre programmes, ticket stubs) (Mooney, Beale, et al., 2018), and the use of a “daily schedule” index card or a PPA card (Rogalski & Khayum, 2018).

In mild PPA, where cognitive abilities are relatively preserved, the use of non-verbal strategies can also facilitate communication. These may include the use of smartphone applications (e.g., Pic Collage) (Rogalski & Khayum, 2018; Volkmer et al., 2020), rate and rhythm control (Beber et al., 2018), intonation (Volkmer, Walton, et al., 2023), the use of a picture visual aid (Rogalski & Khayum, 2018), and in the case of stuttering in nfvPPA, repositioning the lips and tongue (Douglas, 2021). In cases of severe PPA, non-verbal strategies may include gesturing (Dial & Henry, 2023; El-Wahsh et al., 2021; Fried-Oken et al., 2015; Jokel & Meltzer, 2017; Mooney et al., 2022; Rebstock & Wallace, 2018; Volkmer et al., 2020), pointing (Kindell et al., 2013; Volkmer, Mistry, et al., 2023), and direct, face-to-face attention (Cycyk & Wright, 2008). Additionally, individuals may benefit from sitting near those who engage in excessive verbalization, as this can help to mitigate the effects of language-led fatigue (Davies & Howe, 2019). The use of a signal to mark the end of a turn (Volkmer, Walton, et al., 2023) is another possible strategy. The enactment may be regarded as a verbal (the utilization of direct reported speech) or non-verbal (the deployment of prosody and body movements) strategy and is similarly pertinent in both naturally developed and formally trained contexts (Davies & Howe, 2019; Kindell et al., 2013; Volkmer et al., 2021; Volkmer, Mistry, et al. 2023).

Partner adaptation strategies encompass a range of techniques, from topic orientation (Cycyk & Wright, 2008; Volkmer, Walton, et al., 2023) to positive reinforcement (El-Wahsh et al., 2021), partner-assisted scanning (Fried-Oken et al., 2015), and the modification of question structures using single-question prompts rather than dichotomous options (Cycyk & Wright, 2008). Additionally, these strategies entail providing pwPPA with ample time to express themselves (Dial & Henry, 2023; El-Wahsh et al., 2021; Volkmer et al., 2021), repeating information back to the PPA subject with elaboration (Taylor et al., 2014), and offering options to ascertain the target word (Volkmer, Mistry, et al., 2023). Further examples of partner adaptation strategies can be found in Table 2.

The environmental modification strategies that have been identified in the included studies are the establishment of simple alerting systems, the minimization of distractions, the provision of sticky notes, and the placement of communication aids in functionally appropriate locations, amongst others.

It is important to note that some of the aforementioned strategies are employed by the individual with PPA, while others are utilized by their partner, caregiver or family member.

Tables 1 and 2 present a comprehensive enumeration of the communication strategies identified as being used within the context of pwPPA everyday life.

**Table 1.** Deatiled Description of the Verbal and Non-verbal Commucation Strategies Used in PPA

**Verbal communication strategies**	
• Using any adequate word when the perfect word is not available • Using “thing” when the target word is not available • Giving additional information around a person’s identity when unable to generate the name; • Engaging in brief conversations with strangers to practice • Making notes of keypoints beforehand • Mentally rehearse relevant vocabulary or sentences • Writing letters in the air • Writing keywords or messages on a paper • Using self-cueing strategies to improve word access (semantic and orthographic or first syllable/phoneme self-cueing) • Using or emphasizing key words • Using script aids (reading written scripts or expressing a memorized script)	• Responsive naming • Memory books or memo boards • Communication book • Communciation wallet • E-mail • Lightwriter • Yes/no eye blinks • Using prompt cards • Texting • Using automatic phrases in conversations • Description of the target word • Enactment (use of direct reported speech) • Using of confirm/reject/repetition • Using remnants • Requesting assistance from the partner or asking for help for word retrieval
• Speech generating devices (to produce oral messages)	
• Voice recognition technology (to write)	
• Smartphone applications (e.g., notes, ARCUS, CoChat)	
• Using a “daily schedule” index card, or a PPA card	
• Using self-repair with revision strategies	
**Non-verbal communication strategies**	
• Rate and rhythm control	• Tongue clicks
• Face-to-face attention	• Eye movements/eye gaze to point
• Sitting next to talkative people	• Gesturing
• Enactment (use of prosody, body movements)	• Mime
• Distraction techniques in conversations (tapping fingers)	• Drawing pictures
• Selecting activities involving speech to avoid fatigue	• Facial expressions
• Periods of silence before important conversations	• Body posture
• When stuttering, reposition lips and tongue	• Smartphone applications (e.g., Pic Collage)
• Vocalizations	• Using pictures on Photo Stream
• Pointing	• Using a picture visual aid
	• Using a signal to mark the end of a turn
	• Using intonation

**Table 2.** Deatiled Description of the Partner Adaptation Strategies and Environmental Modification Strategies Used in PPA

**Partner adaptation strategies**	
• Face-to-face attention/eye contact • Topic orientation • Continuity of topics • Overcoming communication blocks • Question structures: asking single questions rather than either/or questions • Interaction encouragement • Direct types of verbal messages • Reduce sentence length and structure • Minimize complexity of vocabulary • Collaboration - being a conversation support • Elaboration - extending the conversation • Tone of voice • Time allowed for turn-taking • Gesturing • Positive reinforcement • Choose the best time of day for a conversation • Downshifting strategy to prompt a response • Partner-assisted scanning • Using augmented input technique: providing a written choice strategy, written word cues, supplementing spoken language with gestures to facilitate comprehension, written words or phrases, drawings, or diagrams • Using high-frequency vocabulary • Giving plenty of time to speak • Using and modeling multimodal communication strategies	• Collecting remnants from daily activities (e.g., props, objects) to support conversation • Using confirming/rejecting hypothesis set by PPA subject • Request for confirmation or more information • Repeating information back to PPA subject with elaboration • Prompting, cueing and generating specific clues • Supplying additional information that provide cognitive support by providing factual details (e.g., giving information when needed, providing the missing word, using memory, organization suppors, responding to errors by giving correct information in a non-punitive manner, affirming and verifying PPA’s contribution to the conversation) • Giving more time • Waiting for the PPA individual production before offering prompts to help • Avoiding finishing PPA individual’s sentences • Asking less test questions • Letting the person with PPA lead conversations • Using shorter sentences • Asking questions to prompt the person with PPA to choose a topic • Scaffolding • Offering options in an attempt to guess the target word
**Environmental modification strategies**	
• Minimizing distractions • Setting up alerting systems • Environmental signage	• Smartphone applications (e.g., notes, Pic Collage, ARCUS, CoChat) • Video chat
	• Computer-based systems
• Implementing alphabet boards	• On-screen keyboard and joystick to access computer use
• Providing communication books	• Turning off the TV/radio when having conversations
• Making sticky notes available	• Placing communication aids in functionally appropriate locations.
• Calendar	
• Planner	
• Notepad	
• Pocket-sized notebook	

### Effectiveness of Communication Strategies

#### Effectiveness of Communication Strategies in PPA

Research studies reporting communication strategies employed by individuals living with PPA have emphasized the significance of semantic cueing in prompting word retrieval during conversations in their early stages (Cycyk & Wright, 2008). Furthermore, the employment of assistive augmented communication strategies by people living with PPA and their respective partners has been shown to facilitate effective communicative interactions (Fried-Oken et al., 2015) that become multimodal (Mooney, Bedrick, et al., 2018; Volkmer, Mistry, et al., 2023), as well as independence in ADLs (Volkmer et al., 2020) and, consequently, overall quality of life. The low-tech AAC to which reference is made in the included studies is: (i) the utilization of memory books or written reminders (Lanzi et al., 2017); (ii) emphasizing keywords (Jokel & Meltzer, 2017; Volkmer, Walton, et al., 2023); (iii) writing graphemes, keywords and messages (Fried-Oken et al., 2015; Jokel & Meltzer, 2017; Mooney et al., 2022; Roberts et al., 2022; Volkmer, Walton, et al., 2023); (iv) drawing (Fried-Oken et al., 2015; Jokel & Meltzer, 2017; Mooney et al., 2022; Rebstock & Wallace, 2018; Volkmer, Walton, et al., 2023); (v) using signage (Lanzi et al., 2017); (vi) using gestures, facial expressions, enactment (Jokel et al., 2017; Mooney et al., 2022; Volkmer et al., 2021; Volkmer, Mistry, et al, 2023); (vii) using written reminders (Lanzi et al., 2017); (viii) using a calendar and/or a planner (Lanzi et al., 2017); (ix) using a notepad and/or notebook (Lanzi et al., 2017); (x) using written cues combined with picture cues (Lanzi et al., 2017). It has been demonstrated that, particularly, the employment of gesture and enactment are successful coping mechanisms for anomia in functional communication (Davies & Howe, 2019; Volkmer et al., 2021). In addition, the high-tech AAC reported in the records includes the use of applications such as CoChat (Mooney, Bedrick, et al., 2018). The findings of the retrieved studies indicate that the effective use of AAC requires adequate training directed towards both pwPPA and CPs (Fried-Oken et al., 2012). Multimodal turn-construction practices, which also require training, facilitate the co-construction of narratives (Volkmer, Mistry, et al., 2023).

In svPPA, enactment was found to be a good way of establishing functional communication (Davies & Howe, 2019; Kindell et al., 2013) and email helped svPPA individuals to maintain contact with family and friends. Email allowed svPPA to take the time they needed to process the information they were receiving or giving (Davies & Howe, 2019).

In nfvPPA, dysfluency appears to be minimized by repositioning the lips and tongue to the opposite oral position to that intended (Douglas, 2021). Individuals with co-occurring apraxia of speech appear to benefit from rate and rhythm control techniques (Beber et al., 2018) and the use of AAC devices (Nickels & Croot, 2014). The quality and length of communicative exchanges seem to improve with the use of hand gestures and phonological cueing (Cadório, Figueiredo, Martins, Cardoso, et al., 2019; Douglas, 2021). In these patients, fatigue and overstimulation may be avoided by allowing periods of silence before significant conversations and/or limiting the amount of conversation people with nfvPPA engage in (Douglas, 2021). In contrast, semantic cueing and word-finding exercises seem to play an irrelevant role in nfvPPA (Cadório, Figueiredo, Martins, Cardoso, et al., 2019; Douglas, 2021).

In lvPPA, the use of semantic and orthographic/phonemic self-cueing strategies did not improve word retrieval (Grasso et al., 2019).

Other communication strategies have proved less effective. For example, adaptative mechanisms used by family members, such as multiple questions or test questions. These seem to cause frustration and feelings of incompetence in pwPPA (Volkmer et al., 2021). In addition, gestures and drawing are not recommended if the aim is to facilitate word retrieval or change of communication modality (Rebstock & Wallace, 2018). The use of speech-generating devices (e.g., Lightwriter, GoTalk Express 32, Lingraphica) does not support conversational exchanges (Mooney et al., 2022), nor does any other AAC device without formal training (Fried-Oken et al., 2012).

Table 3 provides a summary of the data extraction, including study demographics, communication strategies addressed and their value for communication effectiveness.

**Table 3.** Summary of the Data Extracted From the Full-Text Analysis

Author(s) Year	Study location	Type of study	Sample size	Aim	Population	Communication strategies/techniques	Study outcomes (strategies effectiveness)
Beber et al., 2018	Brazil	Case report	1	To investigate the effects of rate and rhythm control approaches in the treatment of apraxia of speech in nfvPPA.	nfvPPA	Techniques of rate control (patient was trained to lengthen the first sound/syllable of words when difficulty was present) and rhythm control (the therapist provided a rhythm)	The speech strategies might benefit patients with nfvPPA.
Cadório, Figueiredo, Martins, & Lousada, 2019	Portugal	Case report	1	To determine which strategy is more effective in facilitating word retrieval in nfvPPA.	nfvPPA	Semantic strategy (responsive naming) and phonological strategy (the therapist provided the first syllable)	The patient responded better to the phonological stratregy when compared to the semantic strategy.
Cycyk & Wright, 2008	N/A	Review	N/A	To discuss management strategies for patients with frontotemporal dementia.	FTD	Errorless and effortless learning methods; memory training; educational approaches and experiential exercises; group activities; caregiver training (i.e., FOCUSED); memory books. FOCUSED programme consists of seven strategies: face-to-face attention, topic orientation, continuity of topics, overcoming communication blocks, question structures, interaction encouragement, and direct types of verbal messages.	Semantic cueing and word retrieval may be helpful in the initial stages. FOCUSED strategies provide the individual with dementia with communicative opportunities. Using memory books may increase the quality and length of communicative exchanges, as increased informativeness and frequency of utterances was found on nursing home residents who used memory books.
(Davies & Howe, 2019)	Canada United Kingdom USA	Scoping review	N/A	To summarize the qualitative research on the experience of living with PPA from the perspective of the individuals with the disorder and their family members.	PPA	Sitting next to people who like to talk so the person with PPA does not have to talk as much. Enactment strategy involving depicting/performing one’s or others’ talk or thoughts through the use of direct reported speech, prosody, and/or body movement. Using a smarthphone application (ARCUS) and e-mail.	Enactment allowed an individual with svPPA to communicate more effectively with others during conversations. E-mail helped an individual with svPPA to maintain contact with other people, as it offers enough time to understand what one is writing or reading.
Douglas, 2021	USA	Case report	1	To describe the development and implementation of an evolving set of targeter strategies and adaptations designed to enhance the quality of life of a person in the early stages of PPA.	nfvPPA	Word-finding exercises involving flash cards or sentence completion; use of distraction techniques in conversations (e.g., closing eyes, twirling the right index finger, rubbing the ear or tapping fingers); use any adequate word instead of seeking the perfect word; use the word “thing” to substitute the target word; ask for help in seeking a word by providing clues; making running lists of synonyms on index cards; engaging in brief conversations with a stranger when visiting the grocery store; being selective about the activities involving speech that one chooses to engage in; having periods of silence before important conversations; making notes of keypoints beforehand and mentally rehearse relevant vocabulary or sentences; when stuttering, reposition lips and tongue.	Word-finding exercises did not seem to be an advantage, as they were simplistic and encompassed not relevant words to patient’s everyday life. The use of hand gestures helped to cue and accompany the speech. Prioritizing within activities involving speech helped to maximize the ability to communicate in the sense that less is more. Limiting the ammount of conversations per day and experiencing periods of silence before conversations successfully avoided fatigue and overstimulation. The silent repositioning of lips and tongue into the exact opposite oral posture helped to break cycles of repeated dysfluent sounds. Patient’s perception of her own quality of life has improved with the described strategies and adaptations, in association with injections of magnesium sulfate.
El-Wahsh et al., 2021	N/A	Overview	N/A	To provide an outline of the communication changes expirienced in dmentia and an overview of assessment and intervention options for individuals with dementia and their caregivers.	PPA	Training communication partners to reduce sentence length and structure and complexity of vocabulary, collaboration—working together in conversations, elaboration—extending the conversation, to use tone of voice, time allowed for turn-taking, eye contact, gesturing, positive reinforcement, to minimize distractions, choosing the best time of day for a conversation.	Communication partner training programs reported improved quality of life and communication effectiveness.
Fried-Oken et al., 2012	USA	Case series	30	To examine differences in conversational performance of adults with AD related to the presence and absence of an aid, the type of symbol embedded in the aid, and the presence or absence of voice output.	AD	Using an Alternative and Augmentative Communication (AAC) device during structured conversations, sometimes associated with voice output. Using a downshifting strategy to prompt a response (the researcher gradually provides a little more information about the response that is being solicited).	Providing an AAC device to an individual with moderate AD does not necessarily lead to its use in conversation. Instead, the use of AAC requires training to become an assistive tool.
Fried-Oken et al., 2015	N/A	Review	N/A	To present AAC interventions for patients with neurodegenerative diseases affecting speech, motor, language and cognitive domains.	ALS PPA Classical AD	Using no-tech AAC: yes/no eye blinks, vocalizations, tongue clicks, eye movements, writing letters in the air, gestures, and partner-assisted scanning. Using low-tech AAC: writing messages on a paper, drawing pictures, implementing alphabet boards, communication books, simple alerting systems, communcation partners can use a written choice strategy or enhance comprehension by supplementing spoken language with gestures, written words or phrases, drawings, or diagrams (augmented input technique). Using high-tech AAC: on-screen keyboard and joystick to access computer use, computer-based systems, speech-generating devices.	AAC should be standard practice for adults diagnosed with neurodegenerative disease, as a way to maintain effective and functional communication interactions. AAC helps patients to remain engaged in daily activities.
Grasso et al., 2019	USA	Case report	4	To investigated the effects of lexical retrieval treatment administered by both a speech-language pathologist and a caregiver, to two participants with anomia caused by neurodegenerative disease.	Mild cognitive impairment lvPPA CPs	Using self-cueing strategies (semantic and orthographic/phonemic self-cueing) to improve word-retrieval.	Treatment effects were similar for clinician- and caregiver-administered treatment phases. Besides, treatment gains were greatest for trained items. Naming performance remained stable from pre-test to post-test.
Jokel & Meltzer, 2017	Canada	Comparison-group study	20	To provide a structured and comprehensive group intervention for individuals afflicted with PPA and their spouses.	PPA Spouses	Emphasizing keywords, writing them down, drawing, using gestures, and facial expressions.	Facility with communication strategies used by dyads increased pre- to post-treatment. PPA individuals showed successful utilization of strategies when relaying messages to their spouses.
Dial & Henry, 2023	N/I	Overview	1	To illustrate the principles of assessment, diagnosis, and intervention in PPA by describing a case study.	PPA	Learning to use a picture book with functional items grouped by category and paired with written words, as a conversation support. Partner traning focused on emphasizing ways to maximize and support communication: using high-frequency vocabulary, giving plenty of time to speak, and using and modeling multimodality communication strategies (e.g., writing, drawing, and gesturing).	N/I
Kindell et al., 2013	UK	Case report	1	To examine the everyday conversation, at home, of an individual with semantic dementia.	svPPA	Repeated practice of acting out different scenes (enactment), using direct reported speech along with paralinguistic features (such as pitch and loudness) and non-vocal communication (such as body posture, pointing and facial expression).	The use of enactment allows the individual with severe svPPA to convey information more effectively than if he communicated events verbally. Enactment strategy also helps the patient to better perform important interactional goals, such as take his turn and progress the conversation.
Lanzi et al., 2017	USA	Review	N/A	To outline the continuum of dementia severity and the cognitive-communication characteristics at each stage as a guide for selecting compensatory communication supports to fit changing needs.	PPA AD Neurocognitive disorders	Using external strategies, such as: written reminder; memory books; calendar; planner; memo board; notepad; pocket-sized notebook; sticky note; written cues combined with picture cues (simple declarative sentences -one per page- and a relevant photograph/illustration); having a designated place for an object; reading written scripts or expressing a memorized script; environmental signage. Caregiver training (e.g., teaching caregivers to use multimodal communication approaches).	The use of external strategies assisted the retrieval of personal information necessary to maintain conversations. Patients were able to elicit elaborated comments about the topic, reduce the frequency of ambiguous and repetitive verbalizations, increase turn-taking, maintain the topic, and reduce partner prompting and conversational dominance. The use of signage supported independence and wayfinding (e.g., locating a room) of nursing home residents. Teaching specific communication strategies can promote enhanced communication in the home environment.
Mooney, Bedrick, et al., 2018	USA	Case series: multiple baseline design	10	To develop and implement a PPA group treatment for diagnosed subjects and communication partners.	PPA CPs	Using augmented input strategies (written word cues provided by conversation partners, key-wording, written choice. Using wallet cards and communication books. Collecting remnants from daily activities to use as content for conversation. Using scripts (monologues and dialogues). Using mobile technology and built-in apps. Capitalizing on multimodal communication support.	People with PPA and communication partners reported increased use of multimodal communication.
Mooney, Beale, & Fried-Oken, 2018	USA	Single-subject alternating treatments experimental design	6	To examine the treatment effects of a novel language compensation tool (CoChat) on lexical retrieval skills during activity retell.	PPA	Using CoChat application, an AAC iOS-platform application that generates lexical displays on a tablet based on user-captured photos, related comments, and an automatically curated list of target words.	CoChat led to improved word retrieval in a natural conversational context. PPA individuals found conversations to be easier when using this technology.
Mooney et al., 2022	USA	Single-blind, randomized, parallel group, active-control, behavioral clinical trial	41	To characterize the use, frequency, and perceived effectiveness of communication modes reported by individuals with PPA.	Mild to moderate PPA	Using facial expression, gestures, writing/drawing, nonelectronic AAC, apps on a smart phone/tablet, speech-generating devices, texting, video chat (e.g., zoom, skype), e-mail.	Perceived effectiveness of communication modes used by persons with mild-to-moderate PPA was higher for texting, writing/drawing and nonelectronic AAC and lower for speech-generating devices.
Nickels & Croot, 2014	N/A	Review	N/A	To review current understanding of PPA and challenges that remain to be addressed in future research, and to direct readers to additional sources of information that will allow them to gain further knowledge about topics of interest.	PPA	Using AAC, such as a photo communication book or a lightwriter.	AAC devices can be of great assistance to people with PPA, particularly in the advanced stages and/or when co-occurring apraxia of speech or mutism.
Rebstock & Wallace, 2018	USA	Case report	1	To examine the effects of a combined semantic feature analysis and multimodal communication program on word-retrieval accuracy, switching among modalities, and overall communicative effectiveness.	PPA	Gesturing and drawing.	There were no consistent changes in word-retrieval accuracy or switching communication modalities after Semantic Feature Analysis combined with multimodal treatment.
Roberts et al., 2022	USA	Randomized control trial	180	To determine the efficacy of Communication Bridge-2 intervention for individuals with mild to moderate PPA.	PPA CPs	Communication Bridge web application encompassing training scripts, word-retrieval cueing hierarchy (phonological-semantic), use of multi-modal communication supports (e.g., using layered and deployed communication modes - gestures/body language, writing, texting, drawing, and speaking). Building communication alliances to scaffold successful communication. Using technology solutions (e.g., picture or story board web applications for conveying information or retelling events, speech-to-text and text-to-speech native apps).	N/I (Project in course, yet to conclude).
Rogalski & Khayum, 2018	USA	Review	N/A	To outline the current literature on the diagnosis and intervention options directed to PPA. To describe the principles of the Communication Bridge Care Model.	PPA	Using speech-generating devices to produce oral messages and voice recognition technology to write. Using apps on smart phones or tablets (ex. “notes” section, Pic Collage). Using pictures on Photo Stream, a picture visual aid, a communication wallet, and/or remnants. Using a “daily schedule” index card. Using a PPA card or a PPA script aid. Turning off the TV/radio when having conversations, placing communication aids in functionally appropriate locations.	The Communication Bridge Care Model has been shown to be feasible for individuals living with mild to moderate PPA using a telemedicine approach.
Taylor et al., 2014	Australia	Parallel group, active-control, behavioral clinical trial	12	To describe the repair behaviours used by the individuals with PPA and their communication partners.	nfvPPA e lvPPA CPs Controls	PPA: using self-repair with revision strategies, using of confirm/reject, using repetition, requesting assistance from the partner in situations of lexical retrieval difficulties. Partner: using confirming/rejecting hypothesis set by PPA subject, request for confirmation or more information, repeating information back to PPA subject with elaboration, supplying the missing word.	Some repair/revision strategies were successful and some others not. Individuals with PPA were able to maintain turn-taking and contribute to the social interaction, but their partners bore the greater responsability of highlighting need for repair using collaborative, interactive trouble-indicative behaviours.
Taylor et al., 2017	Australia	Parallel group, active-control, behavioral clinical trial	28	To investigate the conversational productivity and the trouble and repair behabiours in people with svPPA compared to controls.	svPPA CPs Controls	PPA: repetitions with request for specific information Partner: prompting, cueing, supplying additional information that provide cognitive support by providing factual details (e.g., giving information when needed, using memory, organization suppors, responding to errors by giving correct information in a non-punitive manner, affirming and verifying PPA’s contribution to the conversation).	The better the naming ability, the greater the percent success of repairs occurred in conversation. Success of repair strategies varied from dyad to dyad.
Volkmer et al., 2020	N/A	Review	N/A	To bring together current approaches to managing PPA, highlighting the barriers to access to specialist speech and language therapy and suggests future priorities for developing better care.	PPA	Gesture; use of assistive augmentative communication devices (high and low technology - smartphones and communication books)	Assistive augmentative communication devices that employ both high and low technology can support ADLs (e.g., shopping, cooking and conversations with trained conversation partners).
Volkmer et al., 2021	UK	Review	N/A	To develop a manual and an online training resource for speech and language therapists to deliver a communication partner training program called Better Conversations with PPA.	PPA	PPA: using gestures and enactment (whole body gesture and pantomime). Partner: giving more time.	The use of gesture and enactment by a person with PPA may be an effective coping strategy in the face of difficulties in retrieving a spoken word. Adaptative mechanisms used by conversations partners, such as multiple questions or test questions may result in frustration for the person with PPA, making her/him feel incompetent.
Volkmer, Mistry, et al., 2023	UK	Single-blind randomized controlled pilot study	36	To pilot the Better Conversations with PPA program compared to a control group that receive a different treatment.	PPA CPs Controls	PPA: using gestures when encoutering word finding difficulties or to indicate understanding, giving additional information around a person’s identity when unable to generate the name, using prompt cards to facilitate conversations, using eye gaze to point to indicate word/topic, hand gesturing/mime, choosing the topic, using keywords, using a gesture/signal to mark the end of turn, using writing and drawing in conversations, using automatic phrases in conversations, using intonation. Partner: waiting for the PPA individual production before offering prompts to help, avoiding finishing PPA individual’s sentences, asking less test questions, letting the person with PPA lead conversations, asking single questions rather then either/or questions, using shorter sentences, asking questions to prompt the person with PPA to choose a topic, using props/objects to support conversations.	The Better Conversations with PPA program has the potential to prevent the evolution of poor mental health for people with PPA and their communication partners.
Volkmer, Walton, et al., 2023	UK	Case report	4	To investigate collaborative turn-construction practices of people with PPA and their partners.	lvPPA mixed PPA CPs	PPA: gesturing, mime, pointing, facial expression, enactment, description of the target word. Partner: scaffolding, generating specific clues, offering options in an attempt to guess the target word/word.	Multimodal turn-construction practices from PPA individuals coupled with partners’ collaboration in naming what they enact, successfully allow the co-construction of a narrative.

## Discussion

The implementation of strategies and approaches designed to optimize communication is of great relevance to pwPPA, who experience a detriment to their quality of life as they lose their premorbid speech level of function (Douglas, 2021). However, there is a dearth of information regarding which strategies facilitate effective communication in PPA in comparison with post-stroke aphasia. In contrast to post-stroke aphasia, which has been characterized by a more stable presentation, PPA is recognized as a progressive condition. Consequently, there has been an ongoing attempt to review, devise, and implement appropriately targeted strategies and adaptations when difficulties with speaking, reading, or writing arise (Douglas, 2021). The aim of this review was to identify the communication strategies employed and/or trained in PPA and to ascertain their value to functional communication.

The key to assisting pwPPA in dealing with the challenges they face daily is to teach CPs meaningful skills and strategies to improve communication (Burgio et al., 2001; O’Rourke et al., 2018). In communicative interactions, the literature shows that partner assistance is more helpful than prompting or cueing (Taylor et al., 2014). CPs might acquire the ability to utilize communication strategies through formal instruction. Said instruction may include such activities as collaborative problem-solving, role-playing, strategy practice activities with performance feedback, and conjoined reflections, in addition to other therapeutic activities (Roberts et al., 2022). In this regard, Volkmer and colleagues (Volkmer et al., 2020) state that speech-language therapy should provide CPs training to maximize opportunities to build on, reinforce and generalize existing communication strategies in a relevant context. The delivery of CPs’ training is typically conducted by the speech and language therapist or pathologist. The ‘Better Conversations with Primary Progressive Aphasia’ (BCPPA) programme, for example, has been developed to facilitate improved communication in pwPPA and respective CPs. The programme was designed to reduce barriers to conversation and increase facilitators to conversation through the use of participant’s pre-intervention video-recorded conversation samples, to provide clips for video feedback during intervention sessions (Hoffmann et al., 2014).

Similar to the evidence from post-stroke aphasia, building on existing communication skills may be a more effective way of compensating for communication deficits than learning new strategies (Volkmer et al., 2020). In the process of selecting strategies or techniques, it is imperative to consider the preserved language abilities of pwPPA. These include phonological and autobiographical memory processes in svPPA, and semantic memory abilities in nfPPA and lvPPA (Cadório, Figueiredo, Martins, & Lousada, 2019; Croot, 2018). To leverage patients’ strengths, it is recommended that a variety of communication modalities be employed (Fried-Oken et al., 2015). The integration of AAC through assistive tools, environmental adaptations, and behavioural modifications is more readily embraced when CPs are engaged from the initial stages of the disease. Multimodal communication aids, when scaffolded by CPs, appear to complement both receptive and expressive language skills, thereby increasing communicative effectiveness (King & Simmons-Mackie, 2017), increasing social participation and improving decision making (May et al., 2019; Rebstock & Wallace, 2018). It is important to note that strategies may be subject to modification over time to achieve positive outcomes in the face of gradual deterioration in language ability (Fried-Oken et al., 2015).

A communication-supportive environment helps a person know what to expect, encourages appropriate and functional behaviours, uses multiple modalities to educate, and minimizes distracting stimuli (Brush et al., 2012).

## Conclusion

In PPA, person-centered, functional goals should focus on developing automatic, efficient use of communication strategies that avoid cognitive burden on the individuals diagnosed with PPA and their CPs.

Training CPs is as important as creating an environment for optimal communication (e.g., turning off the TV during conversations, placing communication aids in functionally relevant locations, making sticky notes available). Often, individuals diagnosed with PPA and their immediate social circle have already developed their own strategies for everyday conversations. In such cases, the speech and language therapist’s role is to identify which strategies are working well, to encourage the continued use of these strategies, to support generalization of these strategies to personally relevant settings, to identify communication patterns that may act as barriers, to offer additional strategies in anticipation of impending decline, and to adjust the use of strategies within the course of the disease (Rogalski & Khayum, 2018).

This scoping review shows that PPA population use verbal and non-verbal communication strategies, environmental communication strategies and partner adaptation strategies to facilitate successful communication. To summarize, the employment of assistive augmented communication strategies, comprising both low-tech and high-tech AAC, appears to enhance communication effectiveness. This is attributable to the utilization of multimodal resources, which aid in compensating for progressive language decline.

Future research should investigate which strategies better serve the patients’ needs at each time point, taking into consideration the PPA subtype.

## Supplemental Material

Supplemental Material - Communication Strategies Used in Primary Progressive Aphasia: A Scoping Review Supplemental Material for Communication Strategies Used in Primary Progressive Aphasia: A Scoping Review by Inês Cadório and Daniela Vieira in Dementia

## Appendix

### Appendix I

**Table 4.** Reasons for Exclusion of Records at Both the Screening Stage (*n* = 225) and the Full-Text Analysis Stage (*N* = 3)

Number of records	Reasons for exclusion
*N* = 211	Does not address the use/training of communication strategies
*N* = 2	It was not possible to access the record.
*N* = 9	The manuscript was about pos-stroke aphasia and/or other conditions that did not include PPA
*N* = 5	The manuscript was unclear about the type of aphasia (vascular-based or neurodegenerative-based)
*N* = 1	The manuscript was a research protocol
