# Supplementary figures and images for: Communication Strategies Used in Primary Progressive Aphasia: A Scoping Review
Source: Dementia (London). 2025 Jul 9;25(4):899–924. doi: 10.1177/14713012251356588 (PMC13062470; doi:10.1177/14713012251356588)

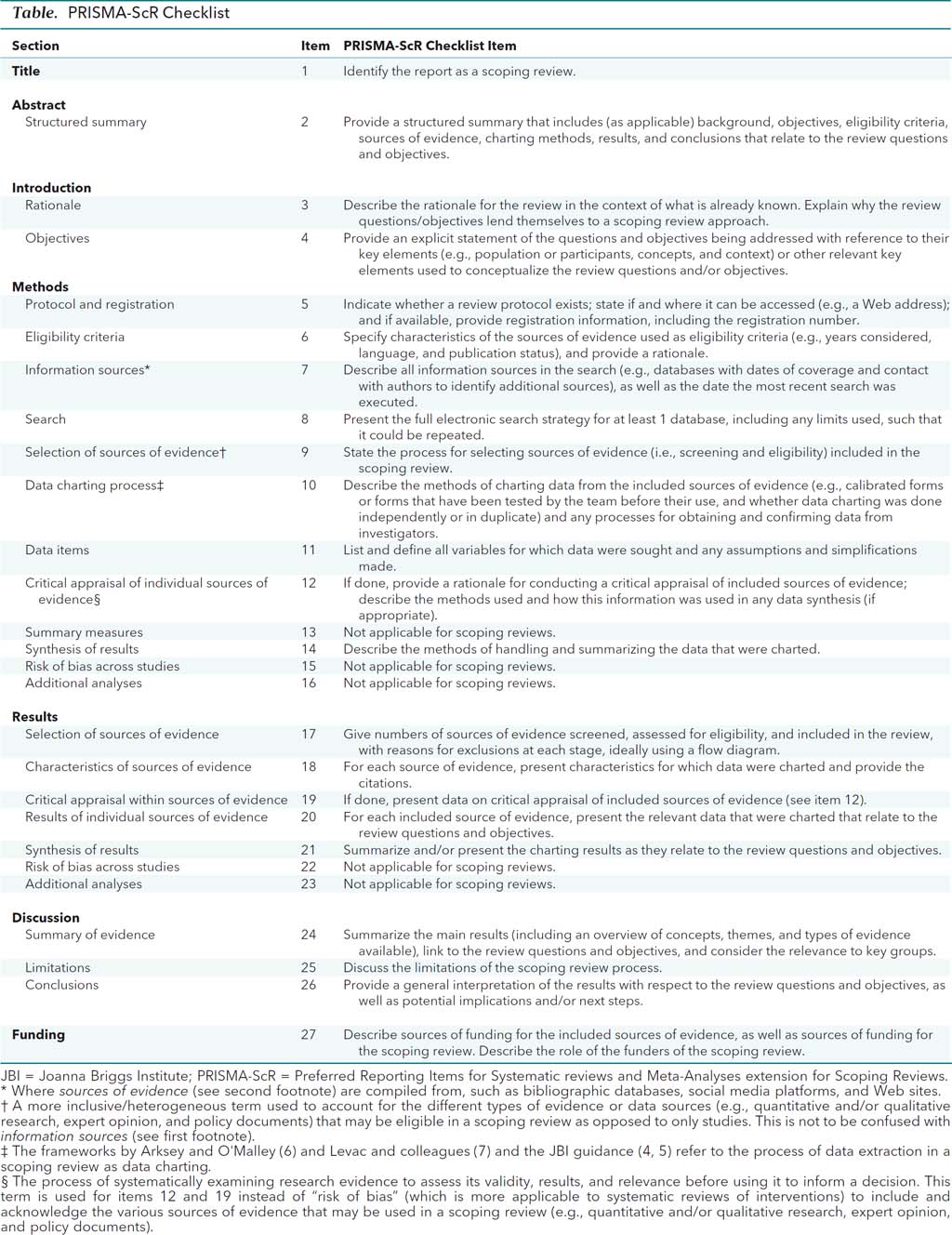

Supplement: Supplemental Material - Communication Strategies Used in Primary Progressive Aphasia: A Scoping Review [file sj-jpg-1-dem-10.1177_14713012251356588.jpg]
